# Supplementary figures and images for: How Do Runners Experience Personalization of Their Training Scheme: The Inspirun E-Coach?
Source: Sensors (Basel). 2020 Aug 15;20(16):4590. doi: 10.3390/s20164590 (PMC7472115; doi:10.3390/s20164590)

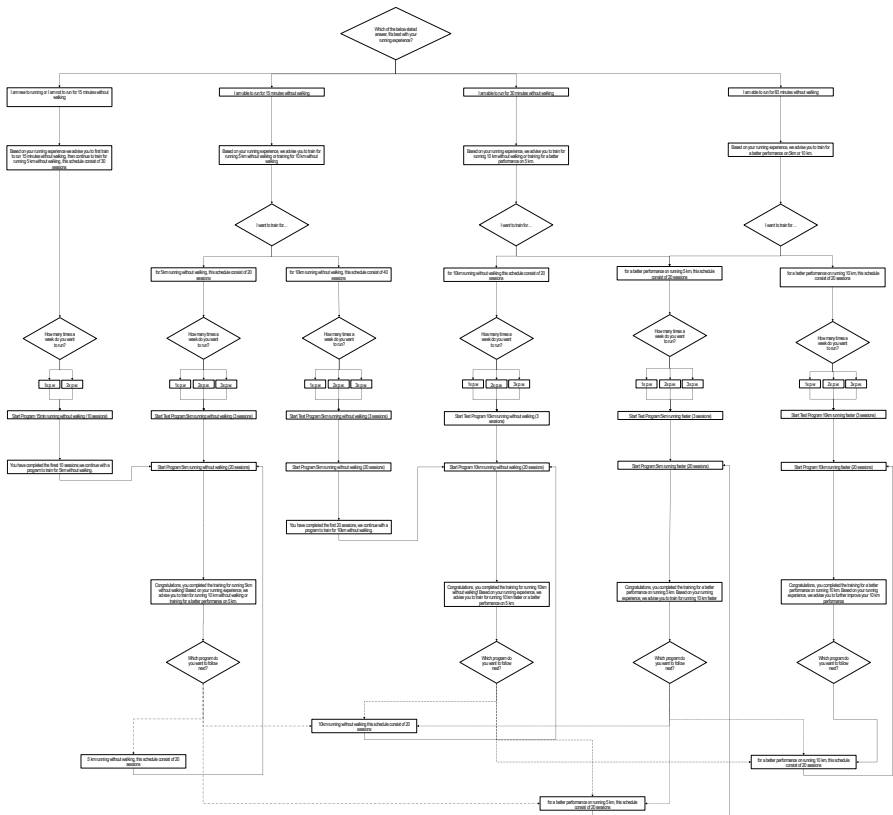

Supplement: Supplementary file 1 [file sensors-20-04590-s001.pdf]
